# Circulating cellular communication network factor 1 (CCN1) as a liquid biopsy marker indicating progression in advanced melanoma

**DOI:** 10.1186/s12967-026-07724-y

**Published:** 2026-01-21

**Authors:** Isabel Heidrich, Kim-Lea Reese, Helen Ullemeyer, Julian Kött, Hanna Freiberg, Glenn Geidel, Alessandra Rünger, Inga Hansen-Abeck, Finn Abeck, Stefan W. Schneider, Christoffer Gebhardt, Klaus Pantel, Daniel J. Smit

**Affiliations:** 1 Institute of Tumor Biology, University Medical Center Hamburg-Eppendorf, Martinistraße 52, 20246 Hamburg, Germany; 2 Department of Dermatology and Venereology, University Medical Center Hamburg-Eppendorf, Martinistraße 52, 20246 Hamburg, Germany; 3 Fleur Hiege Center for Skin Cancer Research, University Medical Center Hamburg-Eppendorf, Martinistraße 52, 20246 Hamburg, Germany

**Keywords:** Melanoma, Cellular communication network factor 1, CCN1, Immune checkpoint inhibition, Prognostic biomarker, Liquid biopsy

## Abstract

**Background:**

Cellular communication network factor 1 (CCN1, also referred to as CYR61), a secreted matricellular protein, has been implicated in tumor progression and stromal remodeling within the metastatic tumor microenvironment of melanoma. Here, we investigated, for the first time, whether CCN1 circulating in the blood can serve as a biomarker in melanoma patients.

**Methods:**

In this retrospective study, serum CCN1 levels before treatment initiation were measured by enzyme-linked immunosorbent assay (ELISA) in 95 patients with advanced melanoma (unresectable AJCC stage III and AJCC IV) treated with immune checkpoint inhibitors. The association between CCN1 serum levels and clinico-pathological parameters, as well as clinical outcomes, was analyzed using Kaplan-Meier survival curves and Cox proportional hazards models. Moreover, CCN1 levels were also evaluated in relation to established biomarkers, including S100B.

**Results:**

An optimal cutoff of 221.76 pg/mL was calculated for serum CCN1 to stratify patients into high and low CCN1 groups. No significant associations, despite T status, with demographic, clinico-pathological, or laboratory parameters of the CCN1 groups were detected. High serum CCN1 levels were significantly associated with reduced OS (median OS: 15 months vs. median OS not reached, *p* = 0.011), but only a trend was toward impaired PFS was detected. Combination of CCN1 with established prognosticators in melanoma, such as S100B serum levels, enhances risk stratification. Patients with high serum levels of both CCN1 and S100B exhibited the poorest prognosis (median OS: 5 months), while those with low levels of CCN1 and S100B had the most favorable outcomes (median OS not reached; overall log-rank *p* < 0.0001, adjusted *p* = 0.00032), indicating the complementary value of CCN1. In the multivariate Cox-regression analysis, CCN1 sustained as an independent prognostic factor of impaired OS (HR = 3.50, 95% CI: 1.69–7.26, *p* = 0.001) besides Eastern Cooperative Oncology Group (ECOG) performance status 2 (HR: 4.10, 95% CI 1.62–10.36, *p* = 0.003) and elevated S100B (HR: 4.64, 95% CI: 1.93–11.16, *p* = 0.001).

**Conclusion:**

CCN1 is an independent prognostic blood-based liquid biopsy biomarker for OS in advanced melanoma (especially if combined with S100B), suggesting a potential role in melanoma aggressiveness and potential involvement in immunotherapy resistance that warrants further functional investigation.

**Supplementary Information:**

The online version contains supplementary material available at https://doi.org/10.1186/s12967-026-07724-y.

## Introduction

Malignant melanoma is a highly aggressive skin cancer, accounting for more than 330,000 new cases and more than 50,000 deaths worldwide per year [1]. Since the introduction of immune checkpoint inhibitor (ICI) therapy for advanced melanoma, survival rates have increased tremendously [2]. However, despite the increasing survival rates, a substantial number of patients still experience disease progression [3]. Especially for immunotherapy, it has been shown that the tumor microenvironment plays an important role in therapy efficacy and response [4]. Among the matricellular proteins implicated in these processes, cellular communication network factor 1 (CCN1, also known as CYR61) has emerged as a potential modulator of tumor progression, angiogenesis, and stromal remodeling in solid tumors including melanoma [5–7].

Our group has previously shown that circulating CCN1 has been proposed as a novel blood-based biomarker for the detection of breast and lung cancer [8, 9], suggesting its utility in non-invasive cancer diagnostics. Furthermore, CCN1 is expressed in disseminated breast cancer cells [8, 10], suggesting a potential role in cancer metastasis. In melanoma, expression of the CCN family member CCN1 can be detected in tumor tissues, including tumor cells and cancer-associated fibroblasts (CAFs) [5]. Functional studies have demonstrated that high CCN1 levels are associated with enhanced vascularization, cell migration, and invasive behavior of tumor cells, underscoring its role as a pro-metastatic effector in melanoma progression and metastatic outgrowth [11–13].

Circulating proteins have become valuable biomarkers used in “liquid biopsy” analysis of blood and other body fluids [14]. Although CCN1 has been implicated in various malignancies, its clinical relevance as a blood-based circulating biomarker in melanoma is unexplored. In particular, its value as a prognostic biomarker and its interaction with established prognostic melanoma markers, such as S100B, have not been systematically studied [15, 16]. Therefore, this study aims to evaluate the prognostic significance of circulating CCN1 in patients with advanced melanoma. We assess the association of serum CCN1 with clinical outcomes in patients receiving immunotherapy.

## Materials and methods

### Study population

This retrospective study included 95 patients with histologically confirmed advanced melanoma American Joint Committee on Cancer (AJCC) unresectable stage III and IV who received ICI either in combination with CTLA-4 inhibitor Ipilimumab (3 mg/kg body weight) and PD-1 inhibitor Nivolumab (1 mg/kg body weight every 3 weeks) or either Nivolumab monotherapy (480 mg every 4 weeks) or Pembrolizumab monotherapy (200 mg every 3 weeks) at the Department of Dermatology and Venereology, University Skin Cancer Center Hamburg of the University Medical Center Hamburg-Eppendorf. Patients with melanoma were enrolled between February 2018 and April 2025. All patients provided informed consent to participate in this study, which was approved by the Ethics Committee of the Hamburg Chamber of Physicians under the number PV5392.

### Peripheral blood processing

Peripheral blood samples were collected prior to therapy initiation (baseline). Blood was collected into 7.5 ml serum gel tubes (S-Monovette^®^ 7.5 ml Sarstedt, Germany) and centrifuged for 10 min at 1800 x *g* to isolate serum within 2 h after the blood draw. Serum was stored at -80 °C until further analysis.

### Enzyme-linked immunosorbent assay (ELISA) for CCN1 levels

The quantitative determination of CCN1 levels in the serum of melanoma patients was performed using the CCN1 enzyme-linked immunosorbent assay (ELISA) kit, according to the manufacturer’s instructions (R&D Systems, Catalog No. #DCYR10). Briefly, Wells were filled with 100 µL of assay diluent RD1-36. Thereafter, 50 µL of standards of CCN1 recombinant protein and serum samples were added to pre-coated 96-well plates with the CCN1 antibodies and incubated for 2 h at room temperature on a horizontal orbital microplate shaker. Thereafter, the wells were washed four times with the supplied wash buffer. Next, 200 µL of the supplied anti-CYR61 antibody conjugated with horseradish peroxidase was added and incubated for 2 h on a horizontal orbital microplate shaker. Following the incubation, again, the wells were washed four times with the supplied wash buffer. 200 µL of substrate solution was added to well and incubated for 30 min in the dark at room temperature. Then, 50 µL of stop solution was added to each well. Absorbance was measured at 450 nm and 570 nm using a microplate reader (Power Wave XS2, BioTek, Winooski, VT, USA). Absorbance values at 570 nm were subtracted from the values at 450 nm before generating the standard curve. Concentrations were calculated from the linear regression model based on the standard curve generated on each plate after log/log transformation. All standards and samples were measured in duplicates. Clinical samples were randomly distributed among the plates blinded to clinical data.

### Analysis of other blood parameters

Thrombocyte as well as, leucocyte (incl. neutrophils, eosinophils, basophils, lymphocytes) counts were measured in peripheral blood by routine clinical laboratory analysis using an ADVIA^®^ 2120i System analyzer (Siemens). The neutrophil/lymphocytes (NLR) ratio was calculated based on the absolute numbers of neutrophils and lymphocytes. LDH was measured by spectrophotometry (Atellica Solution CH930), S100 by chemiluminescence (Liaison XL), and CRP by turbidimetry (Atellica Coag 360 and Atellica Solution CH930, respectively). D-dimer levels were measured in serum directly after blood draw using Innovance D-Dimer Assay (Siemens AG, Munich, Germany) on BCS XP System (Siemens AG, Munich, Germany). The following thresholds were applied to define the parameters as elevated: LDH: 245 U/L; S100B: 0.152 µg/mL. S100B and CRP values below the lower limit of detection (LoD) were included as metric values with half of the lower limit of detection (LoD S100B: 0.021 µg/mL; LoD D-dimer: 0.19 mg/L; LoD CRP: 5 mg/L).

### Statistical analysis

For data analysis and visualization, the following packages were used in RStudio (R version: 4.4.1): *ggplot2* version 3.5.1, *finalfit* version 1.0.8, *survminer* version 0.5.0, *survival* 3.8.3, and *maxstat* version 0.7.25. Routine laboratory measurements extracted from patient records below the lower limit of detection were set to equal half of the lower limit of detection for analysis of metric variables. Categorical variables were analyzed using Fisher’s exact test. All continuous variables were assessed for normal distribution using the Shapiro-Wilk test. In cases where normal distribution was present, equality of variance was tested using the Levene test. Parametric data are reported as mean ± standard deviation (SD). In contrast, non-parametric data are reported as median ± interquartile range (IQR). The means of continuous variables were compared either using Student’s t-test (parametric data of two groups with equal variance), Welch’s t-test (parametric data of two groups with unequal variance), or the Mann-Whitney U test (non-parametric data), where applicable. The optimal cut-off point for dichotomization in CCN1 groups was determined using the *maxstat* package based on overall survival (OS) as an outcome. Survival curves were plotted using the Kaplan-Meier method, and the statistical significance of differences in survival times with respect to OS and progression-free survival (PFS) was analyzed by the log-rank test (Mantel-Cox). Cox Proportional Hazard Regression analysis was used to determine predictors of OS and PFS in univariate and multivariate models. Parameters that were significant prognosticators in univariate analysis were included in multivariate analysis. A *p*-value of < 0.05 was considered statistically significant.

## Results

We analyzed serum-derived CCN1 Protein levels in 95 melanoma patients with unresectable stage III and stage IV melanoma, as classified by the American Joint Committee on Cancer (AJCC). The mean age of melanoma patients at diagnosis was 61 years (SD 16.7). Of these, 30.5% were female (*n* = 29) and 69.5% were male (*n* = 66). Regarding histological subtypes, 76.8% (*n* = 73) of patients had cutaneous melanoma, 7.4% (*n* = 7) had mucosal melanoma, and 15.8% (*n* = 15) had a histologically confirmed melanoma of unknown primary (MUP). Tumor (T) classification showed that 21.1% of patients had a T0, 9.5% T1, 13.7% T2, 10.5% T3, 43.2% T4 cancer, whereas 2.1% were not accessible (Tx). Regarding lymph node involvement (N), 33.7% of patients were N0, 21.1% were N1, 16.8% were N2, 25.3% were N3, and 3.2% were not accessible (Nx). Most patients (94.7%) had distant metastases (M1), whereas 5.3% of patients were either mucosal melanoma without distant metastasis or other histological subtypes with unresectable tumors. In terms of therapy, most patients (72.6%) received a combination of CTLA-4 and PD-1 inhibitors, while 27.4% received PD-1 inhibitor monotherapy. Most patients (71.6%) were treated in the first-line setting, while 22.1% received second-line therapy and 6.3% more than 2 therapy lines. Regarding therapy responses according to the RECIST criteria, 11.6% of patients achieved a complete response (CR), 15.8% a partial response (PR), 20% stable disease (SD), and 52.6% showed progressive disease (PD). The baseline concentration of the biomarker CCN1 was available for all patients, with a mean value of 287.3 pg/mL (SD: 209.3 pg/mL). An overview of the demographic, clinico-pathological parameters of the study cohort can be found in Table 1.

**Table 1:** Demographic, clinical and pathological characteristics of the study population

	Total *N* (%)	Levels	*N* (%)
Age at diagnosis	95 (100.0)	Mean (SD)	61.0 (16.7)
Sex	95 (100.0)	Female	29 (30.5)
		Male	66 (69.5)
Primary melanoma site	95 (100.0)	Cutaneous	73 (76.8)
		Mucosal	7 (7.4)
		MUP	15 (15.8)
Best overall response	95 (100.0)	CR	11 (11.6)
		PD	50 (52.6)
		PR	15 (15.8)
		SD	19 (20.0)
AJCC	95 (100.0)	Stage III	3 (3.2)
		Stage IV	92 (96.8)
T	95 (100.0)	T0	20 (21.1)
		T1	9 (9.5)
		T2	13 (13.7)
		T3	10 (10.5)
		T4	41 (43.2)
		Tx	2 (2.1)
N	95 (100.0)	N0	32 (33.7)
		N1	20 (21.1)
		N2	16 (16.8)
		N3	24 (25.3)
		Nx	3 (3.2)
M	95 (100.0)	M0	5 (5.3)
		M1	90 (94.7)
ECOG	95 (100.0)	0	57 (60.0)
		1	24 (25.3)
		2	10 (10.5)
		3	3 (3.2)
		4	1 (1.1)
*BRAF* mutational status	85 (89.5)	Wildtype	54 (63.5)
		Mutated	31 (36.5)
*NRAS* mutational status	72 (75.8)	Wildtype	51 (70.8)
		Mutated	21 (29.2)
Therapy regimen	95 (100.0)	CTLA-4 + PD-1	69 (72.6)
		PD-1	26 (27.4)
Therapy line	95 (100.0)	First line	68 (71.6)
		Second line	21 (22.1)
		Third line	4 (4.2)
		Fourth line	2 (2.1)
CYR61 at baseline [pg/mL]	95 (100.0)	Mean (SD)	287.3 (209.3)

AJCC stages were encoded according to the 8th edition of the AJCC cancer staging manual. Cancers of unknown primary were histologically assessed for melanoma origin and referred to as melanoma of unknown primary (MUP). NRAS and BRAF mutational status are based on routine tissue mutational analysis. Abbreviations: AJCC: American Joint Committee on Cancer; CCN1: cellular communication network factor 1; CR: complete response; PD: progressive disease; PR: partial remission; SD: stable disease; T: tumor; N: lymph node; M: distant metastasis; ECOG: Eastern Cooperative Oncology Group; MUP: melanoma of unknown primary

As no standardized cut-off value for CCN1 exists, we have determined the optimal cut-off for prognostication through maximally selected rank statistics with OS time and status as input. Based on the determined optimal cutoff value of 221.76 pg/mL for serum CCN1, patients were stratified into two groups. The low CCN1 group exhibited a median serum concentration of 152.56 pg/mL (interquartile range [IQR]: 129.82–180.60; *n* = 40), while the high CCN1 group showed a median of 346.99 pg/mL (IQR: 252.68-395.41; *n* = 55). Associations between CCN1 levels and both clinico-pathological and laboratory parameters were subsequently evaluated. Demographic, clinical and pathological characteristics were largely comparable between the low and high CCN1 groups, only the T group (*p* = 0.027) was associated with the CCN1 groups (Table 2). Moreover, no statistically significant differences were observed in baseline levels of LDH, S100B, CRP, D-dimers, leukocyte subsets, or the neutrophil-to-lymphocyte ratio (NLR) (Suppl. Table 1).

**Table 2:** Association of demographic, clinical and pathological characteristics of the advanced melanoma cohort according to the CCN1 group

	Total *N* (%)		Low CCN1	High CCN1	*p*-value
Age at diagnosis	95 (100.0)	Mean (SD)	60.0 (18.7)	61.7 (15.1)	0.620
Sex	95 (100.0)	Female	13 (32.5)	16 (29.1)	0.822
		Male	27 (67.5)	39 (70.9)	
Primary melanoma site	95 (100.0)	Cutaneous	31 (77.5)	42 (76.4)	0.153
		Mucosal	5 (12.5)	2 (3.6)	
		MUP	4 (10.0)	11 (20.0)	
Best overall response	95 (100.0)	CR	6 (15.0)	5 (9.1)	0.089
		PD	16 (40.0)	34 (61.8)	
		PR	10 (25.0)	5 (9.1)	
		SD	8 (20.0)	11 (20.0)	
T	95 (100.0)	T0	4 (10.0)	16 (29.1)	**0.027**
		T1	3 (7.5)	6 (10.9)	
		T2	10 (25.0)	3 (5.5)	
		T3	3 (7.5)	7 (12.7)	
		T4	19 (47.5)	22 (40.0)	
		Tx	1 (2.5)	1 (1.8)	
N	95 (100.0)	N0	14 (35.0)	18 (32.7)	0.805
		N1	8 (20.0)	12 (21.8)	
		N2	5 (12.5)	11 (20.0)	
		N3	11 (27.5)	13 (23.6)	
		Nx	2 (5.0)	1 (1.8)	
M	95 (100.0)	M0	2 (5.0)	3 (5.5)	1.000
		M1	38 (95.0)	52 (94.5)	
ECOG	95 (100.0)	0	22 (55.0)	35 (63.6)	0.254
		1	10 (25.0)	14 (25.5)	
		2	5 (12.5)	5 (9.1)	
		3	3 (7.5)	0 (0.0)	
		4	0 (0.0)	1 (1.8)	
*BRAF* mutational status	85 (89.5)	Wildtype	22 (62.9)	32 (64.0)	1.000
		Mutated	13 (37.1)	18 (36.0)	
*NRAS* mutational status	72 (75.8)	Wildtype	20 (71.4)	31 (70.5)	1.000
		Mutated	8 (28.6)	13 (29.5)	
Therapy regimen	95 (100.0)	CTLA-4 + PD-1	29 (72.5)	40 (72.7)	1.000
		PD-1	11 (27.5)	15 (27.3)	
Therapy line	95 (100.0)	First line	33 (82.5)	35 (63.6)	0.096
		Second line	6 (15.0)	15 (27.3)	
		Third line	0 (0.0)	4 (7.3)	
		Fourth line	1 (2.5)	1 (1.8)	

AJCC stages were encoded according to the 8th edition of the AJCC cancer staging manual. Cancers of unknown primary were histologically assessed for melanoma origin and referred to as melanoma of unknown primary (MUP). NRAS and BRAF mutational status are based on routine tissue mutational analysis. CCN1: cellular communication network factor 1; CR: complete response; PD: progressive disease; PR: partial remission; SD: stable disease; T: tumor; N: lymph node; M: distant metastasis; ECOG: Eastern Cooperative Oncology Group; MUP: melanoma of unknown primary

**Fig. 1:**
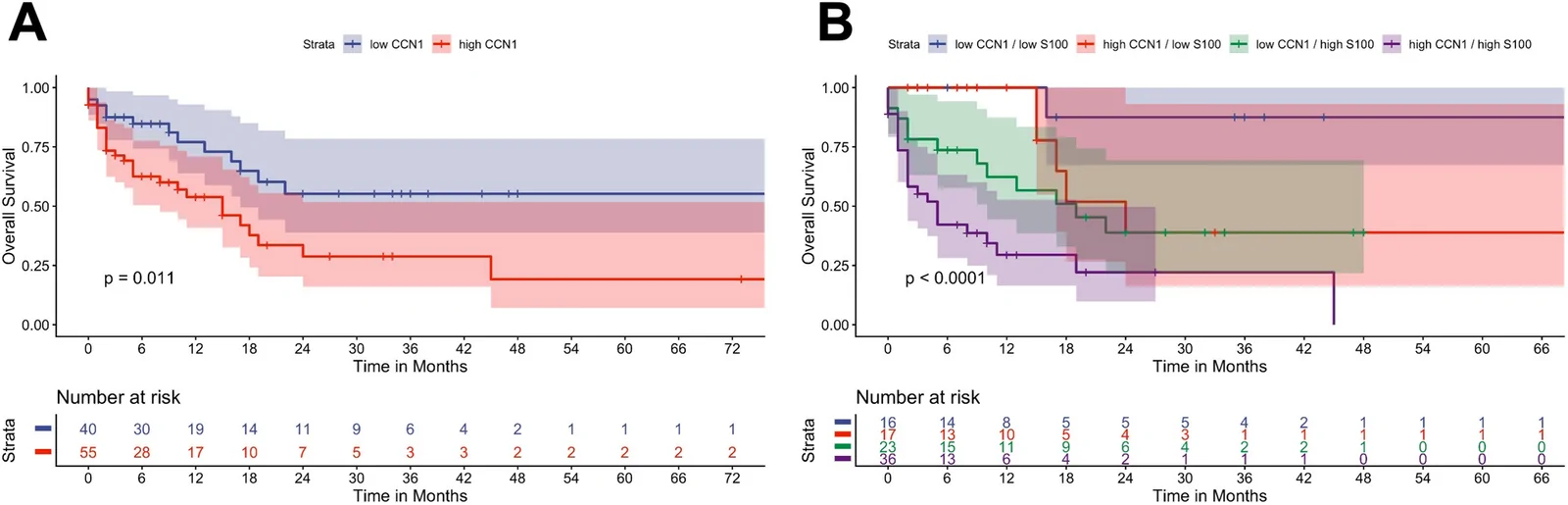
Kaplan-Meier survival analyses (OS) stratified by CCN1 serum levels and the combination of CCN1 with S100B serum levels. **(A)** Kaplan-Meier survival curve showing overall survival (OS) of advanced melanoma patients (*n* = 92) stratified by CCN1 serum levels (low < 221.76 pg/mL vs. high ≥ 221.76 pg/mL) according to the optimal cut-off value. Statistical significance was calculated using the log-rank test (Mantel-Cox). Number of OS events: 13 (low CCN1), 29 (high CCN1); Number of censored patients: 27 (low CCN1), 26 (high CCN1). **(B)** Kaplan-Meier survival curve showing OS of advanced melanoma patients (*n* = 95) stratified by CCN1 serum levels (low vs. high) according to the optimal cut-off value, as well as serum S100B levels. Statistical significance was calculated using the log-rank test (Mantel-Cox), and the overall log-rank p-value is shown. CCN1: cellular communication network factor 1. Number of OS events: 1 (low CCN1 / low S100B), 5 (high CCN1 / low S100B), 12 (low CCN1 / high S100B), 24 (high CCN1 / high S100B); Number of censored patients: 15 (low CCN1 / low S100B), 12 (high CCN1 / low S100B), 11 (low CCN1 / high S100B), 12 (high CCN1 / high S100B)

Next, Kaplan-Meier analyses were performed for both PFS and OS. Patients in the low CCN1 group had a median PFS of 7 months (95% CI: 2–31), compared to 3 months (95% CI: 2–5) in the high CCN1 group. Although a slightly shorter PFS was observed in patients with elevated CCN1 levels, this difference did not reach statistical significance, but indicated a trend towards impaired PFS (*p* = 0.087) (Suppl. Figure 1). In contrast, the analysis of OS revealed a statistically significant difference between the two groups. Patients with low CCN1 levels had a longer median OS with the median OS not reached (95% CI: 17-NA) compared to patients with high CCN1 levels in the blood with a median OS of only 15 months (95% CI: 8-NA) (*p* = 0.011) (Fig. 1A).

To evaluate the combined prognostic value of CCN1 and already established prognostic marker S100B in melanoma, patients were stratified into four groups based on high or low baseline serum levels of both biomarkers. To investigate the complementary prognostic value of CCN1 and S100B, OS was analyzed using combined stratification based on high or low baseline levels of both biomarkers (i.e., CCN1 and S100B) using Kaplan-Meier estimates and log-rank testing. Statistical analysis revealed a significant difference in OS across the groups (overall log-rank *p* = 0.0017) (Fig. 1B). Patients with concurrent high CCN1 (≥ 221.76 pg/mL) and high S100B (≥ 0.152 µg/mL) serum concentrations demonstrated the worst prognosis, with a median OS of 5 months (95% CI: 2–19). In contrast, patients in the low CCN1 (< 221.76 pg/mL) / low S100B (< 0.152 µg/mL) group showed the most favorable long-term outcomes, with median OS not reached (95% CI: NA-NA) during the observation period. Intermediate survival was observed in patients with elevation of only one marker: 24 months (95% CI: 17-NA) in the high CCN1 / low S100B and 19 months (95% CI: 10-NA) in the low CCN1 / high S100B group, respectively **(**Fig. 1B**)**. Pairwise comparisons confirmed that the high CCN1 / high S100B group had significantly impaired OS compared to the low CCN1 / low S100B group (adjusted *p* = 0.00032) and also to the high CCN1 / low S100B or low CCN1 / high S100B group (adjusted *p* = 0.00357 and 0.04351, respectively).

With respect to PFS, again, patients in the low CCN1 / low S100B group demonstrated the most favorable median PFS of 11 months (95% CI: 7-NA), whereas the high CCN1 / high S100B group had the shortest median PFS of only 2 months (95% CI: 1–4) (Suppl. Figure 2). Intermediate outcomes were observed in patients with elevated levels of either CCN1 or S100B (high CCN1 / low S100B: 5 months (95% CI: 3-NA); low CCN1 / high S100B: 2 months (95% CI: 1-NA)). Statistical analysis revealed a significant difference in PFS across the groups (overall log-rank *p* = 0.017). In pairwise comparisons, the most pronounced difference was observed between the low CCN1 / low S100B and the high CCN1 / high S100B group (adjusted *p* = 0.014). No significant differences were observed between intermediate-risk groups after correction for multiple testing.

In a next step, Cox proportional hazard regression analysis was performed to identify independent prognosticators of OS in melanoma patients. In univariate analysis, ECOG 2 (HR: 2.64, 95% CI: 1.12–6.24, *p* = 0.027) was determined as a risk factor for OS. Additionally, elevated S100B levels were significantly associated with an increased risk of death in univariable analysis (HR: 4.88, 95% CI: 2.05–11.60; *p* < 0.001). High serum levels of CCN1 were a moderate risk factor for OS (HR: 2.30, 95% CI: 1.19–4.44, *p* = 0.013). In multivariate analysis, ECOG 2 (HR: 4.10, 95% CI: 1.62–10.36, *p* = 0.003), elevated S100B levels (HR: 4.64, 95% CI: 1.93–11.16, *p* = 0.001) as well as high serum CCN1 levels (HR: 3.50, 95% CI: 1.69–7.26, *p* = 0.001), sustained as independent prognosticators of OS in melanoma patients (Table 3). Age, sex and elevated serum LDH did not significantly impact OS as prognostic markers.

**Table 3:** Univariate and multivariate Cox proportional hazard analysis for overall survival in advanced melanoma patients

Overall survival			HR (univariable) (95% CI)	HR (multivariable) (95% CI)
Sex	Female	29 (30.5)	-	-
	Male	66 (69.5)	0.88 (0.46–1.68, *p* = 0.702)	-
Age at diagnosis	Mean (SD)	61.0 (16.7)	1.01 (0.99–1.03, *p* = 0.193)	-
ECOG	0	57 (60.0)	-	-
	1	24 (25.3)	1.88 (0.93–3.79, *p* = 0.079)	2.30 (1.09–4.87, *p* = 0.030)
	2	10 (10.5)	**2.64 (1.12–6.24**,***p* = 0.027)**	**4.10 (1.62–10.36**,***p* = 0.003)**
	3	3 (3.2)	0.00 (0.00-Inf, *p* = 0.997)	0.00 (0.00-Inf, *p* = 0.998)
	4	1 (1.1)	0.00 (0.00-Inf, *p* = 0.999)	0.00 (0.00-Inf, *p* = 0.999)
S100B	Not elevated	33 (35.9)	-	-
	Elevated	59 (64.1)	**4.88 (2.05–11.60**,***p* < 0.001)**	**4.64 (1.93–11.16**,***p* = 0.001)**
LDH	Not elevated	27 (29.7)	-	-
	Elevated	64 (70.3)	1.56 (0.72–3.41, *p* = 0.261)	-
CCN1 group	Low CCN1	40 (42.1)	-	-
	High CCN1	55 (57.9)	**2.30 (1.19–4.44**,***p* = 0.013)**	**3.50 (1.69–7.26**,***p* = 0.001)**

CCN1: cellular communication network factor 1; CI: confidence interval; ECOG: Eastern Cooperative Oncology Group; LDH: Lactate dehydrogenase

## Discussion

This study provides the first evidence that circulating CCN1 can be detected in sufficient amounts in the blood of melanoma patients. Strikingly, in our cohort, high CCN1 serum levels were strongly associated with poor OS, and the negative prognostic role of elevated CCN1 concentrations was sustained in multivariable Cox regression analysis with a HR of 3.5 among other known prognosticators of melanoma such as S100 and higher ECOG status.

Since all patients in our study received immune checkpoint inhibition treatment, it can be speculated that CCN1 may also contribute to resistance to immunotherapy, however, in this manuscript no functional data are reported. A recent report by Fan et al. demonstrates that CCN1 enhances immunosuppression in pancreatic cancer by repelling T cells in the tumor microenvironment [17], supporting this hypothesis.

Moreover, in our cohort, the prognostic value of CCN1 was increased when analyzed in combination with the established serum biomarker S100B [18, 19] Patients with concurrent high levels of serum CCN1 and elevated serum S100B experienced the worst clinical outcomes for both, OS and PFS, suggesting a complementary effect of these markers. While S100B reflects tumor burden and necrosis [20], CCN1 may contribute complementary information about the tumor microenvironment, such as stromal activation or inflammatory signaling that enhances the prognostic power [21–23]. Thus, our present data suggest that a combined biomarker approach of established melanoma biomarkers and novel liquid biopsy analytes such as CCN1 could enhance risk stratification and identify patients at highest risk for early progression under immunotherapy. In contrast to S100B, LDH as another established tumor marker did not reach statistical significance in our cohort, although our reported HR of 1.56 for elevated LDH was comparable to the findings from Janka et al. reporting on a significant prognostic effect of elevated LDH concentrations with a HR 1.57 [19].

Compared to other liquid biopsy markers such as cell-free DNA (cfDNA) or circulating tumor cells (CTCs), the detection of circulating tumor-associated proteins such as CCN1 may lack high tumor specificity. Still, they could be useful as first-line screening marker indicating progression, which may then lead to more elaborate testing using cfDNA or CTCs. Such a strategy could result in earlier switches to more effective therapies that can be personalized based on the results of the cfDNA or CTC testing [24, 25].

Our present findings highlight the potential translational relevance of CCN1 as an independent prognostic marker in melanoma, consistent with its mechanistic role in promoting angiogenesis, extracellular matrix remodeling, and immune evasion—hallmarks of aggressive tumor behavior [5, 17, 26]. CCN1 exerts its diverse biological functions through binding to various integrins primarily via its conserved TSP-1 and CT domains, which can then activate further downstream signaling pathways [7, 27, 28]. These integrin-mediated interactions enable CCN1 to regulate key cellular processes such as adhesion, migration, proliferation, and senescence (26). The ability of CCN1 to interact not only with integrins but also with other receptors including heparan sulfate proteoglycans, low-density lipoprotein receptor-related proteins, and fibroblast growth factor receptors highlights its broad functional repertoire in modulating cell fate under both physiological and pathological conditions [5, 15, 29, 30]. Although most reports favor the tumor-promoting role of CCN1, others have also reported that CCN1 could act as an anti-tumorigenic protein by inhibiting the cell cycle and increasing apoptosis, indicating that the effect of CCN1 is dependent on the concentration, context, and cellular composition [31].

Despite the promising findings, our study has some limitations. First, it was based on a single-center, retrospective cohort with a moderate sample size, which limits the generalizability of the findings and thus, our results remain exploratory. Second, an external validation cohort which is required to generalize the findings, is not present in this manuscript and therefore, the data should be carefully interpreted. Moreover, in this study, all biomarker measurements were conducted retrospectively at a single time point before treatment initiation, rather than longitudinally. Future studies could address these limitations through a prospective design, longitudinal sampling during ICI treatment, and validation in larger, multicenter cohorts.

## Conclusion

In conclusion, our study identifies CCN1 as a novel, independent prognostic blood-based marker in advanced melanoma. Its complementary value when combined with S100B suggests that a multi-marker approach may offer superior prognostic power. Given its role in crucial cellular processes such as angiogenesis, stromal remodeling, and immunomodulation, CCN1 may also represent a promising therapeutic target and warrants further investigation in pre-clinical and clinical models.

## Supplementary Information

Below is the link to the electronic supplementary material.

Supplementary Material 1

## Data Availability

The data that support the findings of this study are available from the corresponding authors upon reasonable request.

## Abbreviations

CCN1: Cellular Communication Network Factor 1
ICI: Immune Checkpoint Inhibition
LB: Liquid biopsy
AJCC: American Joint Committee on Cancer
T: tumor
N: nodes
M: metastases
IQR: Interquartile range
SD: standard deviation
LDH: lactate dehydrogenase
S100: S100 protein
RECIST: Response Evaluation Criteria in Solid Tumors
MRI: Magnetic resonance imaging
CT: computer tomography
PD: progressive disease
SD: stable disease
PR: partial remission
CR: complete remission
PFS: progression free survival
OS: Overall survival

## Human Genes

BRAF: B-Raf proto-oncogene, serine/threonine kinase
NRAS: NRAS proto-oncogene, GTPase
