# Supplementary material for: Circulating cellular communication network factor 1 (CCN1) as a liquid biopsy marker indicating progression in advanced melanoma
Source: J Transl Med. 2026 Jan 21;24:243. doi: 10.1186/s12967-026-07724-y (PMC12905931; doi:10.1186/s12967-026-07724-y)
Supplement: Supplementary file 1 — Supplementary Material 1 [file 12967_2026_7724_MOESM1_ESM.docx]

**Supplemental Material**

**Suppl. Table 1: Baseline laboratory characteristics of the advanced melanoma cohort according to CCN1 group.**

|  | **Total N (%)** |  | **Low CCN1** | **High CCN1** | ***p*-value** |
| --- | --- | --- | --- | --- | --- |
| **CCN1 at baseline [pg/mL]** | 95 (100.0) | Median (IQR) | 152.555 (129.82 to 180.60) | 346.991 (252.68 to 395.41) | **<0.0001** |
| **LDH [U/L]** | 91 (95.8) | Median (IQR) | 270.500 (223.25 to 394.75) | 328.000 (248.00 to 583.00) | 0.1282 |
| **S100B [µg/L]** | 92 (96.8) | Median (IQR) | 0.258 (0.06 to 0.76) | 0.347 (0.11 to 2.53) | 0.1573 |
| **D-Dimers [mg/L]** | 84 (88.4) | Median (IQR) | 0.760 (0.46 to 1.62) | 1.110 (0.50 to 3.37) | 0.1868 |
| **CRP [mg/L]** | 88 (92.6) | Median (IQR) | 6.000 (2.50 to 19.00) | 14.000 (2.50 to 33.00) | 0.3652 |
| **Thrombocytes [×10^9^/L]** | 93 (97.9) | Mean (SD) | 308.725 (108.54) | 297.321 (140.42) | 0.6597 |
| **Leukocytes [×10^9^/L]** | 93 (97.9) | Mean (SD) | 7.787 (2.70) | 8.557 (2.80) | 0.1838 |
| **Eosinophils [×10^9^/L]** | 33 (34.7) | Median (IQR) | 0.145 (0.11 to 0.25) | 0.120 (0.10 to 0.16) | 0.5634 |
| **Basophils [×10^9^/L]** | 55 (57.9) | Median (IQR) | 0.100 (0.00 to 0.16) | 0.100 (0.00 to 0.14) | 0.7085 |
| **Neutrophils [×10^9^/L]** | 91 (95.8) | Median (IQR) | 4.935 (4.06 to 6.21) | 5.700 (4.38 to 7.37) | 0.1204 |
| **Lymphocytes [×10^9^/L]** | 91 (95.8) | Median (IQR) | 1.480 (1.10 to 1.75) | 1.490 (1.19 to 1.88) | 0.5925 |
| **NLR** | 91 (95.8) | Median (IQR) | 3.099 (2.53 to 4.93) | 3.661 (2.75 to 5.97) | 0.3848 |

**Suppl. Figure 1: Kaplan-Meier survival analyses (PFS) stratified by CCN1 serum levels.** *Kaplan-Meier survival curve showing progression-free survival (PFS) of advanced melanoma patients (n = 95) stratified by CCN1 serum levels (low vs. high) according to the optimal cut off value. Statistical significance was calculated using the log-rank test (Mantel-Cox). Number of PFS events: 26 (low CCN1), 39 (high CCN1); Number of censored patients: 14 (low CCN1), 16 (high CCN1).*

**Suppl. Figure 2: Kaplan-Meier survival analyses (PFS) stratified by CCN1 serum levels and combination of CCN1 with S100B expression levels.** *Kaplan-Meier survival curve showing PFS of advanced melanoma patients (n = 92) stratified by CCN1 serum levels (low vs. high) according to the optimal cut off value as well as serum S100B levels. Statistical significance was calculated using the log-rank test (Mantel-Cox) and the overall p-value is shown. Number of PFS events: 1 (low CCN1 / low S100B), 5 (high CCN1 / low S100B), 12 (low CCN1 / high S100B), 24 (high CCN1 / high S100B); Number of censored patients: 15 (low CCN1 / low S100B), 12 (high CCN1 / low S100B), 11 (low CCN1 / high S100B), 12 (high CCN1 / high S100B).*
